# From the lab to the field with Evolutionary Field Robotics

**DOI:** 10.3389/frobt.2022.1027389

**Published:** 2022-12-05

**Authors:** David Howard

**Affiliations:** Robotics and Autonomous Systems Group, Data61, CSIRO, Brisbane, QLD, Australia

**Keywords:** evolutionary robotics, field robotics, real-world applications, embodied intelligence, morphological computing

## 1 Introduction

This commentary follows on from a recently-published guest editorial in Frontiers in Robotics and AI entitled Evolving Robotic Morphologies (Howard et al., 2022a). Motivated by findings from the many quality submissions, the central thesis of this contribution is that evolutionary robotics and field robotics, despite seeming rather disparate in their application, are in fact closely related by the underpinning theme of a focus on environment. Furthermore, we highlight the potential benefits should the two fields be tied more closely together. Evolutionary Robotics (ER) (Doncieux et al., 2015) applies evolutionary algorithms to create robot behaviours, where behaviour typically emerges through interactions between the robot’s body (e.g., morphology, geometry, sensor and actuator placement), its brain (software controller), and the environment it is assessed in. Experiments are predominantly fitness-driven, where fitness is ascertained from this behaviour. In other words, ER generates solutions – bodies and brains – that are bespoke to the particular environmental niche.

Unlike many fields of research, ER has two distinct goals. Firstly, it has a theoretical goal: to be a reconfigurable experimental apparatus to replicate, understand, and probe biological processes and the mechanisms of natural evolution, with the goal of accumulating knowledge and understanding about those processes. In this regard, ER has been remarkably effective in revealing the purpose and utility behind several mechanisms seen in natural evolution, and as experiments are typically simulated, it is easy to investigate a number of different treatments and draw statistically meaningful conclusions from them. Examples of this type of research include mechanistic implementations of ontogeny (Bongard and Pfeifer, 2003) and environmentally-mediated morphology development (Kriegman et al., 2018), showing how they simplify the “search” process, as well as showing the link between environmental and evolving robot complexity (Auerbach and Bongard, 2014) and inspiring the search for more biologically-rooted evolutionary mechanisms for future instantiation (Hockings and Howard, 2019).

The second goal of ER is applied: to design new types of high performing, environmentally-adapted robots and have them deployed into those environments in reality. The appeal is in creating robots differently to standard engineering approaches, where evolutionary search exploits the close body-brain-environment coupling (Pfeifer and Bongard, 2006) to automatically discover highly bespoke, fit-for-purpose embodiments—robots that no engineer could have conceived of, and robots that perform better than engineered alternatives. It is this goal we focus on herein.

Unfortunately there are very few examples of this happening in reality, although there has been noted success in evolving non-sensing and non-actuating solid geometries (Hornby et al., 2006), modular physical robot composition (Brodbeck et al., 2015), and large projects set up to evolve robots for eventual deployment into nuclear facilities (Hale et al., 2019). To date, and for a plethora of reasons including a lack of high-performance printable materials, the reality gap, and difficulties in body-brain coevolution, evolved physical robots have not been able to consistently outperform off-the-shelf solutions in real environments. Natural evolution abounds with powerful examples of “high performing” creatures that are well-adapted to challenging environmental niches; the ability to create robots with similar traits is therefore an attractive one, and this article proposes a way to progress this research direction.

Unlike ER, Field Robotics (FR) has a singular aim—to develop and deploy rugged autonomous robotic systems into unstructured environments to serve some functional role; be it surveying, agricultural monitoring, ocean floor mapping, or disaster response (Hudson et al., 2021). Field Robotics is an active research area with its own Frontiers Topic Area^1^, and its focus is closer to the secondary focus of ER, i.e., the development of real systems.

Field Robotics systems undergo rigorous evaluation under challenging environmental conditions, and are expected to work reliably and repeatedly in those environments. Field Robotics environments include real terrains, slip, mud, water currents, weather, seasonality, day/night cycles, and so on. FR typically involves interactions within those environments. Development focuses on the use of classical engineering and integration, and testing is primarily done in reality, in a variety of challenging conditions, with emphasis on reliability.

With Evolutionary Robotics and Field Robotics introduced, Evolutionary Field Robotics can be straightforwardly defined as the use of Evolutionary Robotics to provide solutions for Field Robotics problems. For ER, the main transition is from simple (often simulated) environments to complex real environments.

## 2 Evolution, environment, and embodied cognition

Embodied Cognition is a philosophy derived from the study of natural organisms, and postulates that fit (in the Darwinian sense) or high-performing creatures display behaviour that emerges *via* a tight coupling between their environment, their body, and their brain. In other words, the environment is a key determinant of the ability to produce useful behaviour. The key to embodied cognition is ability to interact with environment—o be situated—and many fields across machine learning and robotics are converging on embodied intelligence as a framing device to drive their research into the real world (Roy et al., 2021).

Although they vary greatly in their approach to generating solutions, ER and FR are united in placing significant focus on the role of the environment in their solution-generating processes. With this in mind, it makes sense to view both ER and FR through the lens of Embodied Cognition (Pfeifer and Bongard, 2006), to understand the interplay between the two fields and what could be gained by closer ties between them.

Evolutionary Robotics and Embodied Cognition share a rich history. ER instantiates a form of Embodied Cognition, including delineation of an adaptive body, coupled brain, and assessment in an environment. The environment and coupling mechanism are directly exploited to drive the emergence of useful behaviour. Results are frequently discussed in the language of Embodied Cognition, e.g., *emergence*, *coupling*, and *behaviour*. ER experiments are frequently used to prove theories related to Embodied Cognition, and as such ER can be viewed as a *de facto* algorithmic implementation of Embodied Cognition.

Field Robotics also focuses on the environment, although more as a challenge to be overcome than a driving force behind adaptation. FR does not purposefully exploit Embodied Cognition, although it can be viewed as (frequently control-only) optimisation. Morphological optimisation is not automatic as with ER, but rather a narrower, human-driven search based on engineering principles. FR is bereft of both the inspiration and the vernacular of Embodied Cognition, and the design process does not promote emergence of behaviour. Compared to ER, FR focuses on performance in more demanding, high-fidelity environments that are much closer to the environmental niches found in biological evolution.

## 3 The benefits of Evolutionary Field Robotics

What is to be gained from this merger? The high level answer is that Evolutionary Field Robotics (EFR) brings both fields closer to a natural form of Embodied Cognition, which is known to be a powerful generator of “field-ready” solutions that directly consider the environment.

The crux of the argument is that each element of the body-brain-environment triad must be balanced against the others; the complexity of the controller must match the capabilities of the body and the challenge imposed by the environment (Pfeifer and Bongard, 2006). Raising the complexity and fidelity of the environment promotes the raising of the other two elements to achieve that balance at a higher level. Simulation in ER is typically low resolution. Higher-quality environments allow for more complex embodied interactions to be promoted, leading to higher-fitness robots. The addition of complex real-world environments to ER offers the potential of providing rich, informative learning signals to guide the discovery of tightly coupled body and brain to environment, and drive the discovery of highly capable behaviours to solve these more difficult environments.

This can be attained in three broad steps:

• focus on real “Field” environments,
• make real environmental assessment feasible for ER by increasing simulation accuracy (hybrid data-driven and physics simulation), and
• exploit the scalable, accurate simulator using environment-centric learning techniques already available to ER.

Evolutionary Field Robotics means adoption and focus on the real, challenging environments of Field Robotics. This is a natural progression for ER, that began with simulation-only assessment (Sims, 1994; Cheney et al., 2014). Later, 3D printing allowed for various morphologies to be physically realised (Lipson and Pollack, 2000; Brodbeck et al., 2015; Collins et al., 2018; Howard et al., 2022c) with a view towards real-world deployments (Auerbach et al., 2014; Eiben and Smith, 2015; Hale et al., 2019) and assessment in Field-relevant environmental conditions (Miras et al., 2020).

The consideration of Field problems specifically places the onus on environmental accuracy to instantiate rich information flows between agent and environment and drive the evolutionary process (Nygaard et al., 2021); low fidelity, approximate models wont appropriately capture this behaviour. The physics that governs simulated environments are highly abstracted and simplified. This is particularly detrimental to ER assessment, which typically encompasses a wide range of materials, soft bodies (Mengaldo et al., 2022), rigid and soft environments, and complex contacts (Collins et al., 2021). Although it is known that in some cases abstract representations are enough to promote embodied cognition [e.g., (Narayan et al., 2018)], this simplification may also impose a limit on richness of embodied behaviours that can be simulated. This balance between use of abstract and detailed representations is an open research topic, and one which can be studied through Evolutionary Field Robotics.

The standard Field approach is simply to assess everything in reality, where fidelity is guaranteed and appropriately tight embodiment couplings are encouraged. Conversely, real-world assessment provides this fidelity, but doesn’t scale well when combined with the generational, iterate-and-test approach that underpins ER. Critically for Evolutionary Field Robotics, it will undoubtedly leave an unwanted trail of dead robots in whichever environment it is tested in.

To support the move to richer environments, EFR must develop hybrid approaches combining data-driven modelling with physics simulation. In particular, real data needs to be sent back to the simulator to make it more accurate. Such approaches have been theorised but not fully adopted by ER (Howard et al., 2019; Howison et al., 2021), and pair well with recent advances in Physics-Informed Machine Learning (Karniadakis et al., 2021) to provide accurate, fast simulation of complex physical phenomena.

From the view of Embodied Cognition, Field Robotics does not fully integrate environmental feedback into the design loop. For FR, incorporation of ER serves to shift the fundamental methodology of design towards the natural, emergent intelligence of Embodied Cognition and focuses on tightly coupling robot performance to its environment, including a shift from manual engineering to scalable automated techniques. Further benefits include more consideration for morphology-generating algorithms, which are rarely seen in FR, providing more design freedom in generating solutions and more opportunities to exploit embodiment. Moreover, ER algorithms are black-box in nature, and agnostic to the environment they are tasked to solve. ER can therefore be seen as a general-purpose embodiment generator solver for a range of FR problems and environments to maximise perforamnce and robustness (Carvalho and Nolfi, 2022).

FR can also benefit from the numerous research works investigating how the environment influences both the evolutionary process and evolved robots. Evolution is shown to be tolerant of moderate environmental variations (Milano et al., 2019), and that presenting dynamic environments generates more robust behaviours, matching solution complexity to the difficulty of the environment (Auerbach and Bongard, 2014). Useful environmental mechanisms for FR include Interoceptive signals, which can be used to smooth out complex fitness landscapes, e.g. by modulating mechanical stress directly from environmental interaction (Kriegman et al., 2018). We can also actively shape the learning process through the use of evolutionary curricula (Howard et al., 2022b); options that are not usually a part of the core FR methodology.

## 4 Discussion

Whether autonomously mining on Mars or sustaining deep-ocean surveys, the common long-term vision of applied ER is in solving Field scenarios (Nitschke and Howard, 2021). The main thesis of this article is that is that combining ER and FR, primarily through exploiting richer environmental representations, can create tighter body-brain-environment couplings for the benefit of both fields. As well as providing explicit consideration of Embodied Cognition for Field Robotics, it also provides a route to accelerate technology in applied ER to match the success that theoretical ER has enjoyed to date. By allowing for more expressive environment-robot couplings, theory can also benefit from having more detailed experimental setups with which to probe the fundamental mechanisms of natural evolution.

Of course, this merger is not without it challenges, nor does it solve some common issues that persist in Evolutionary Computing^2^. What it does do, however, is provide a focus on improving the applied outputs of ER, and a route to solving some of the embodiment-related issues that are unique to ER, rather than generically to Evolutionary Algorithms.

To summarise, a focus on Field deployment brings the potential to increase impact by solving real-world tasks, together with corresponding validation of ER as a practical problem-solving tool in the Roboticists’ arsenal.

## Footnotes

1
https://www.frontiersin.org/journals/robotics-and-ai/sections/field-robotics.

2 Including methods to efficiently sample the fitness landscape, setting optimal parameters, and fitness function design, amongst others.
